# Glacial and postglacial sedimentary infill in Slovakian High Tatra Mts. lakes: Acoustic survey and lithological data**

**DOI:** 10.1016/j.dib.2021.107644

**Published:** 2021-11-27

**Authors:** Ramachandran Dhavamani, Radovan Pipík, Valentín Sočuvka, Juraj Šurka, Dušan Starek, Rastislav Milovský, Peter Uhlík, Marina Vidhya, Lucia Žatková, Pavol Král

**Affiliations:** a Earth Science Institute, Slovak Academy of Sciences, Ďumbierska 1, Banská Bystrica SK-97411, Slovakia; b Institute of Hydrology, Slovak Academy of Sciences, Dúbravská cesta 9, Bratislava SK-84005, Slovakia; c Earth Science Institute, Slovak Academy of Sciences, Dúbravská cesta 9, Bratislava SK-84005, Slovakia; d Department of Mineralogy, Petrology and Economic Geology, Faculty of Natural Sciences, Comenius University in Bratislava, Mlynská dolina, Ilkovičova 6, Bratislava SK-84215, Slovakia; e Štátne lesy TANAPu, Tatranská Lomnica SK-059 60, Slovakia

**Keywords:** Chirp sonar, AUV Ecomapper sonar, CT-numbers, Acoustic profiling, Alpine lakes, Pockmarks, Central Europe

## Abstract

The data presented in this paper are related to the research article “Sub-bottom and bathymetry sonar inspection of postglacial lacustrine infill of the alpine lakes (Tatra Mts., Slovakia)” (Dhavamani et al., 2022). An implementation of acoustic sonar protocols provided data for the interpretation of glacigene, glaciolacustrine, postglacial, mass-movement deposits, and geodynamic factors influencing the sedimentation in seven alpine and sub-alpine Tatra Mountains lakes. The field data document the survey track lines of the sonars and allow to identify the location of the geomorphologic phenomena described in (Dhavamani et al., 2022). The laboratory data obtained by micro-CT document the lithology of glaciolacustrine and postglacial lake infill and support the interpretation of sub-bottom sonar record.

**Specifications Table**

**Table:** 

Subject	Earth Sciences, Limnology
Specific subject area	Acoustic inspection of the alpine and sub-alpine lakes (Fig. 1)
Type of data	Video TXT files JSF files Field survey - Core data
How data were acquired	JSF files: Sub-bottom sonar profiler (EdgeTech SB-216S) using Discover SB3200-XS software TXT files: Autonomous Underwater Vehicle EcoMapper (AUVEM – YSI i3XO EcoMapper AUV) Core data: Swimming platform with hydraulic core system UWITEC Niederreiter 60 and gravity corer UWITEC 60 and Micro-CT GE phoenix v|tome|x L240
Data format	Raw Analyzed Transformed
Parameters for data collection	Sub-bottom sonar (SBS) profiler: attached to the front of swimming platform having horizontal to slightly oblique (3°) position against the vessel direction movement; towed 0.4 m below the lake surface; speed of approximately 3.0 m s^−1^; frequency 2–15 kHz for 20 m s^−1^; FM sources 2 kW; ping rate of 6–8 pulses s^−1^ (Fig. 2). AUVEM: system set 500 kHz for depth measurement from 0.5 to 100 m; measurement accuracy ± 0.003 m; Doppler Velocity Log (DVL) navigation sensor. Swimming platform: corer diameter = 60 mm, lengt of one core segment = max. 2 m. Micro-CT: Voltage = 200 kV; Current = 200 µA; Voxel size = 140 µm; Number of images = 800; Timing = 500 ms. The multiscan module was used for the reconstruction of the digital model. The CT data set was analyzed using VG Studio Max 2.2 on a high-end computer workstation.
Description of data collection	The selection of the lakes from Slovakian Tatra Mts. for acoustic sonar surveying were made on previous hydrographic works [2,3] and limited by the conservation restrictions, SBS technical limitation. The SBS profiler was attached to the floating platform and travelled in longitudinal and transverse directions (Fig. 3, Table 1). The data were analyzed for acoustic units that were produced with the SonarWiz® software. The lines with geo-coordinates were exported as tiff images for the creation of chirp sonar expedition route. The AUVEM profiler was used for collecting the bathymetric and topographic data of the lake bottom. Its missions followed a predefined mission plan created in the graphic user environment of the Vector Map Software using GPS waypoints and DVL navigation (Fig. 4) for the collection of data (Table 1). Data analysis was accomplished using Esri's ArcGIS software. Before processing, all data were adjusted and re-projected to follow the vertical datum Bpv (Baltic Vertical Datum - After Adjustment) and horizontal projection S-JSTK (Datum of Uniform Trigonometric Cadastral Network). Digital Elevation Models (DEMs) were created by Geostatistical Analyst tool through the Topo to Raster functionality. Created DEM were inserted to LIDAR slides [4] for correlation of the terrestrial and lake bottom geomorphologic structures. UWITEC sampling platform and corer were applied for coring (Fig. 2; [5] and verification of the acoustic unit ground truth. The cores were
	scanned on micro-CT, sampled for radiometric dating and for the analysis of diatoms, pollens, chironomids, biomarkers, and mineralogical composition. Micro-CT was applied in order to visualize the internal sedimentological structures of core segments and extract information about density and grain-size of the cored lake deposits.
Data source location	Acoustic Sonar data come from 7 Tatra Mts. lakes, Slovakia (Fig. 1, Table 1). SBS profiles were then processed for acoustic units and sedimentary facies. AUVEM bathymetric and topographic data were processed for the identification of lake geomorphological features influencing sedimentation. Missions and acquired SBS and AUVEM data are reported in Figs. 3, 4 and Table 1. Exact location of the lakes is reported in [1]. The cores and samples are stored at the Earth Sciences Institute of SAS in Banská Bystrica (Slovakia) in air-conditioned box under temperature 4,5 °C. The micro-CT experiments were carried out at the Earth Science Institute of the SAS in Banská Bystrica.
Data accessibility	With the article. Raw SBS and AUVEM data collected during fieldwork are deposited at Mendeley Data, https://data.mendeley.com/datasets/7r47wftkgz/2
Related research article	Dhavamani, R., Pipík, R., Sočuvka, V., Šurka, J., Starek, D., Milovský, R., Uhlík, P., Vidhya, M., Žatková, L., Kráľ, P., 2022. Sub-bottom and bathymetry sonar inspection of postglacial lacustrine infill of the alpine lakes (Tatra Mts., Slovakia). *Catena,* 209, 105787. 10.1016/j.catena.2021.105787

**Value of the Data**

• The acoustic and coring data presented in this paper are a result of the 4-years field-survey of the mountain glacial lakes. The acoustic data allow to reconstruct glacigene, glaciolacustrine, and postglacial infill and variability of sedimentary bodies in the lakes. AUVEM data allow to plot bathymetry and bottom topography of lakes.
• The coring and micro-CT data provide support for interpretation of the SBS data in a term of lithology.
• The underwater video conclusively documents the activity of the bottom springs and their role in the completeness of the limnic stratigraphic record.
• These data can interest the researchers investigated sedimentary processes and paleoecological changes in the mountains and mountain lakes.

## 1 Data Description

The data delivered in this paper support the research article entitled “Sub-bottom and bathymetry sonar inspection of postglacial lacustrine infill of the alpine lakes (Tatra Mts., Slovakia).” A detailed acoustic survey supported with lithological data provide information about thickness of Late Pleistocene and Holocene limnic infill [1].

### 1.1 Micro-CT and lithological data

The high-resolution micro-CT images obtained from the sedimentary cores (Fig. 5, Fig. 6, Fig. 7, Fig. 8, Fig. 9, Fig. 10) allowed the identification of sedimentary structures and lake facies that are visible on the SBS profile. X-Ray attenuation of scanned core, expressed as CT-numbers, is extracted using the ImageJ software, which is the function of density, mineralogy, grain-size and sediment porosity. The intensity of the transmitted X-ray beam in the micro-CT is usually expressed as CT-number [6]. The uncalibrated grayscale value (or CT#) in the tiff image is based on how the data were processed while collecting the CT scan. Significant differences in CT numbers in the sediments are visible with respect to the material density (Fig. 6 – POP17-1, section 220–260 cm; Fig. 8 – HIN-1, section 120-150 cm; HIN-2, section 190–230 cm). The clay, silt and coarse grained sediment (gravel, breccia, dropstone) represented by grayscale show higher values whereas the CT-numbers of gyttja show lower values and dark grey color. Black color on CT scans is assigned to gas bubbles (black spots, e.g. Batizovské pleso, Fig. 5), which occur in the deposit due to the decay of organic matter and hence cause the variations in CT-number curve.

For stratigraphic division of the cored lake deposits (Fig. 5, Fig. 6, Fig. 7, Fig. 8, Fig. 9, Fig. 10) we adopted the lithostratigraphic sections recognized on the Polish side of the Tatra Mts. and interpreted in terms of palaeobiological and mineralogical contents [7].

The lower, glaciolacustrine section consists of Late Glacial light grey, fine laminated clay and silt in which can occur layers of sand, gravel or breccia. The section displays low CT-number values from 1900 to 2360 implying the low density contrast and rhythmic alternation of 1,2 mm thick clay and silt laminae which are typical for varves (POP17-1, POP17-2). In case of BAT-1, the CT-number values are lower and vary from 1900 to 2100 (Fig. 5).

The upper, postglacial section consists of brown homogenous gyttja having also low CT-number variations in the lakes with minor clastic input (e.g. Nižné Wahlenbergovo pleso, Fig. 5). The gyttja in POP17-2 and HIN-2 contains numerous clastic layers produced during the extreme episodes (e.g. heavy rain) [8] or periods of intensive rock-failures and debris flow activity [7]. They are visible as significant rise of the CT-number curve. High density dropstones of granitic composition are detected in both the sections displaying very high intensity and also visible as rise of the CT-number curve (light grey to white color).

Zelené pleso Kežmarské is exception in the general lithostraphic division and the postglacial infill thickness. It is infilled only by sand and silt with thickness more than 11 m (Figs. 9 and 10).

### 1.2 Sub-bottom sonar (SBS) data

Raw SBS data collected during fieldwork are published in [9] and can be used for reconstruction of the acoustic images of the lakes. The capital alphabet or number (e.g. A; 1) is denoted as Survey starting point whereas the capital alphabet (e.g. Aʹ; 1ʹ) is denoted as survey ending point (Fig. 3). The acoustic profiles run parallel and then perpendicular to the longitudinal axis of the lake. The survey track lines extracted from the sub-bottom profiles in EdgeTech Discovery software were further imported in SonarWiz 7 as JSF file and these data were then exported as shapefile (.shp – file that stores the feature geometry). The shapefile was inserted in ArcGIS Desktop to overlay it on the satellite imagery base map (Fig. 3).

In Fig. 3, profile highlighted in yellow is the most typical profile interpreted of the given lake. Because the lake record is not uniform in whole lake area, the depicted acoustic profiles show the peculiarities in sedimentary infill and lake bedrock (see also interpreted acoustic profiles in [1]). The SBS acoustic image of Batizovské pleso shows the glacigene deposits overlaid by glaciolacustrine laminated deposit and postglacial gyttja [1], but between the profiles H-Hʹ and G-Gʹ, the sub-vertical line of the weak and chaotic signals interrupts the moderate to high amplitude reflectors of glacigene deposits and this configuration presumes the presence of a tectonic lineage in the granitic basement on the lake outflow. The figure also shows a low-energy backscatter or a very weak to moderate amplitude reflectors with chaotic internal reflections associated with postglacial gyttja with sand admixture sitting directly on glacigene deposits (Fig. 11).

Veľké Hincovo pleso is the fourth largest lake in the Tatra Mts., therefore its bottom topography and lake infill are much variable than in the case of small lakes. The SBS profile C-C′ shows granitic bedrock in the form of isolated mounds surrounded by heavy clastic accumulations of glacigene and rockfall origin [1]. The sub-bottom profile A-Aʹ of Veľké Hincovo pleso shows the glacigene deposits on SSE margin up to depth -20 m below the lake level (Fig. 12). Below this depth, gyttja of the acoustic thickness up to 4 m covers the clastics (Fig. 13), but the core HIN-1 verified only 1.65 m of the lake infill (Fig. 8).

Underwater video (SM1) from Zelené pleso Kežmarské shows the large, elongated conic structure at the bottom and its detail morphology. A moving deposit (time sequence 1:26 min; 1:38 min; 1:45 min on SM1) documents the activity of the bottom springs (pockmarks), permanent mixing of the sedimentary particles, and erosion of the older deposits. Similar structures were confirmed in Batizovské pleso too and their presence can be expected in other Tatra Mts. lakes where the outflow is larger than the inflow.

### 1.3 AUVEM data

Raw AUVEM data collected during fieldwork are published in [9] and can be used for reconstruction of the lake bottom topography. The majority of data was obtained in point form and imported as *x,y,z* files. As the AUVEM records data in WGS84 Geographic Coordinate System (GCS), all data were reprojected to the local vertical datum Bpv (Baltic Vertical Datum - After Adjustment) and the horizontal projection S-JSTK (Datum of Uniform Trigonometric Cadastral Network). Subsequently, these point files were input into the ‘Topo to Raster’ tool as ‘Point Elevation’ data in ESRI ArcGIS, version 10.5 and has been used for the creation of hydrologically-correct DEMs.

## 2 Experimental Design, Materials and Methods

A detailed acoustic sonar survey was carried out on the Slovakian Tatra Mts. lakes from 2016 to 2019 during the late spring and late summer/early autumn periods due to the conservation restrictions, prohibition on using helicopters to transport research materials to barely accessible lakes, and summer storm season in the Tatras. High resolution longitudinal and transversal sub-bottom profiles with a total length of 19 km were captured using EdgeTech SB-216S Chirp Sonar (Figs. 2 and 3; Table 1). For each lake one to three of the most representative profiles are selected to demonstrate the succession of acoustic units and maximal thickness of the lake infill described in [1].

**Fig. 1:**
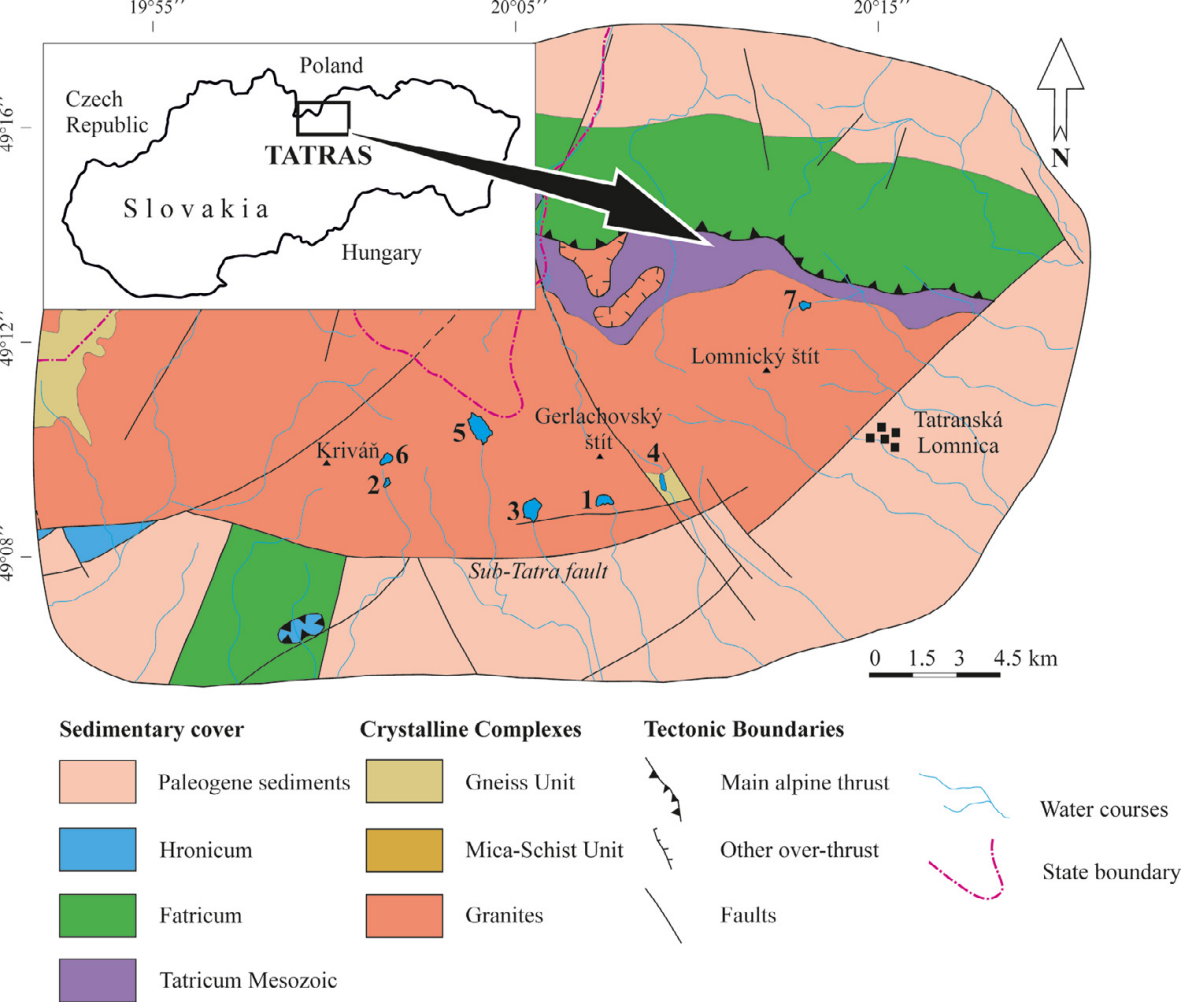
Geological map and location of the studied lakes in the Tatra Mts. [11]; 1 - Batizovské pleso, 2 - Nižné Wahlenbergovo pleso, 3 - Popradské pleso, 4 - Velické pleso, 5 - Veľké Hincovo pleso, 6 – Vyšné Wahlenbergovo pleso, 7 - Zelené pleso Kežmarské.

**Fig. 2:**
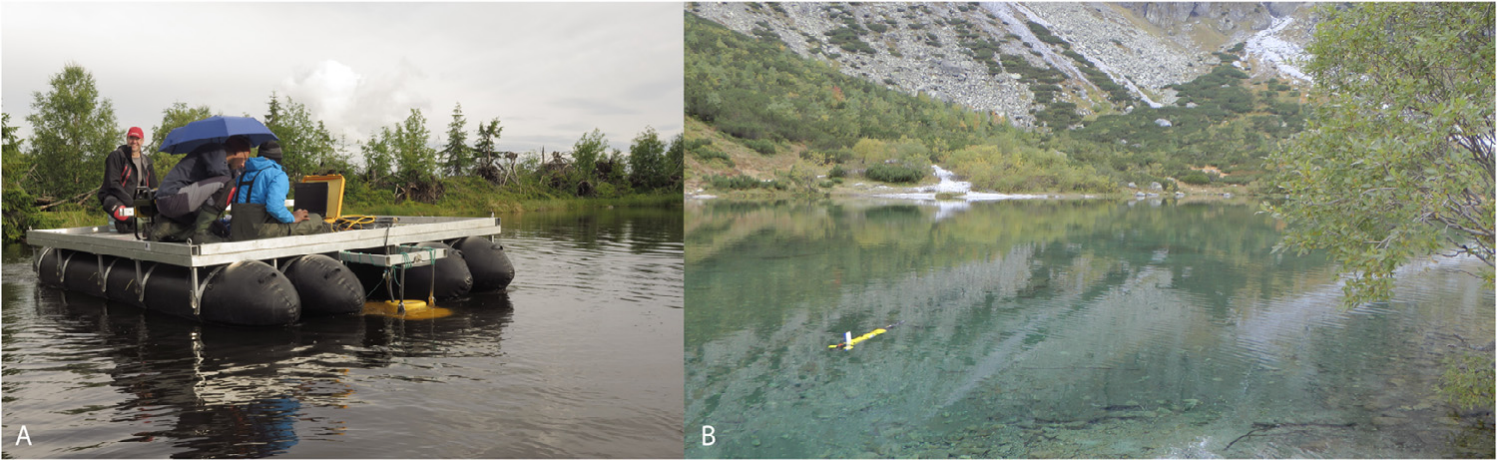
(A) Sub-bottom sonar profiler EdgeTech SB-216S attached to the front of swimming platform having horizontal to slightly obliquely (3°) position against the vessel direction movement (photo R. Milovský). (B) Autonomous Underwater Vehicle YSI i3XO EcoMapper is waiting for signal to start the mission on Zelené pleso Kežmarské (photo V. Sočuvka).

**Fig. 3:**
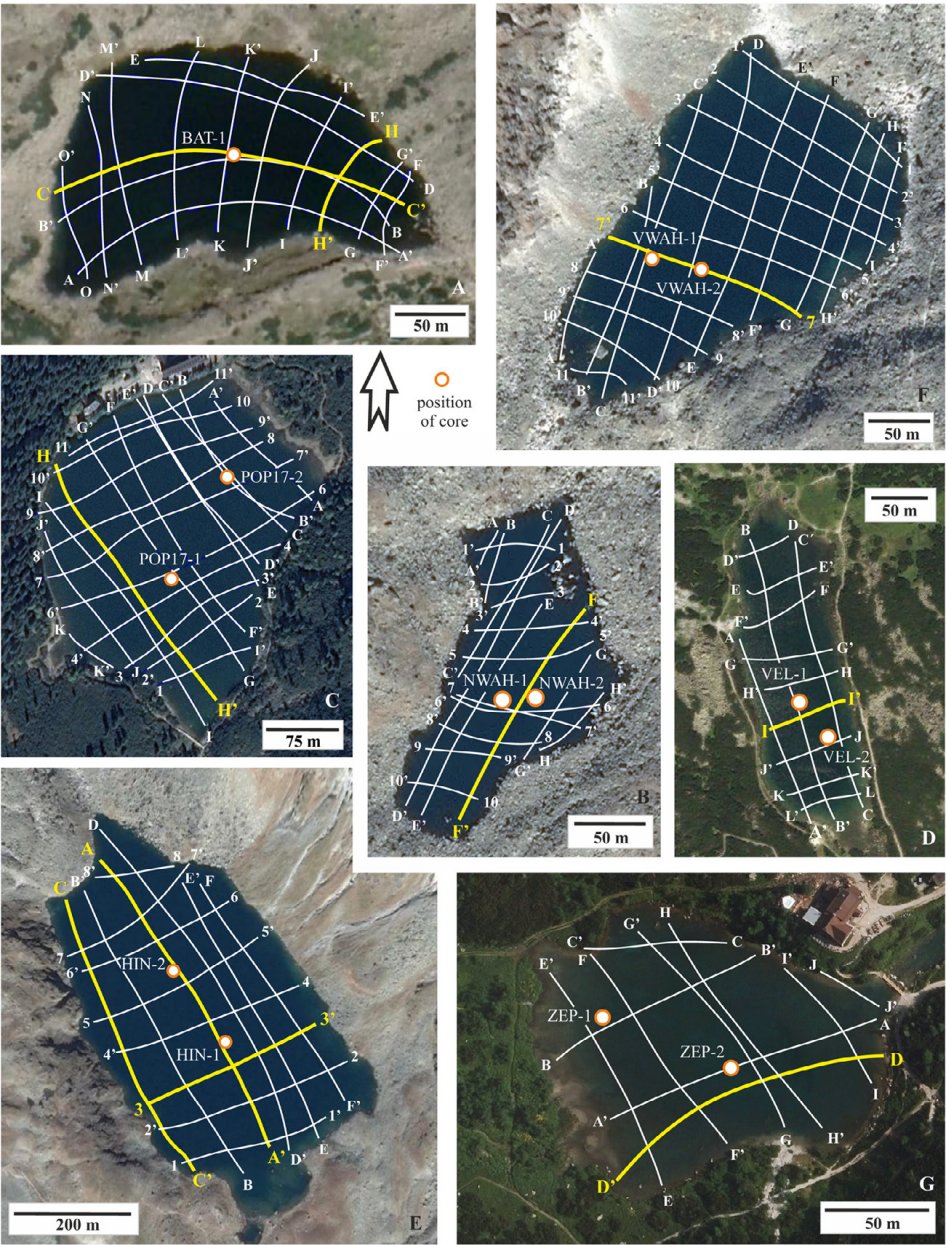
Survey track lines of the sub-bottom profiler. A – survey starting point; A′ - survey ending point. A – Batizovské pleso; B – Nižné Wahlenbergovo pleso; C – Popradské pleso; D - Velické pleso; E – Veľké Hincovo pleso; F – Vyšné Wahlenbergovo pleso; G - Zelené pleso Kežmarské. Yellow line – profiles figured in this article and interpreted in [1].

**Fig. 4:**
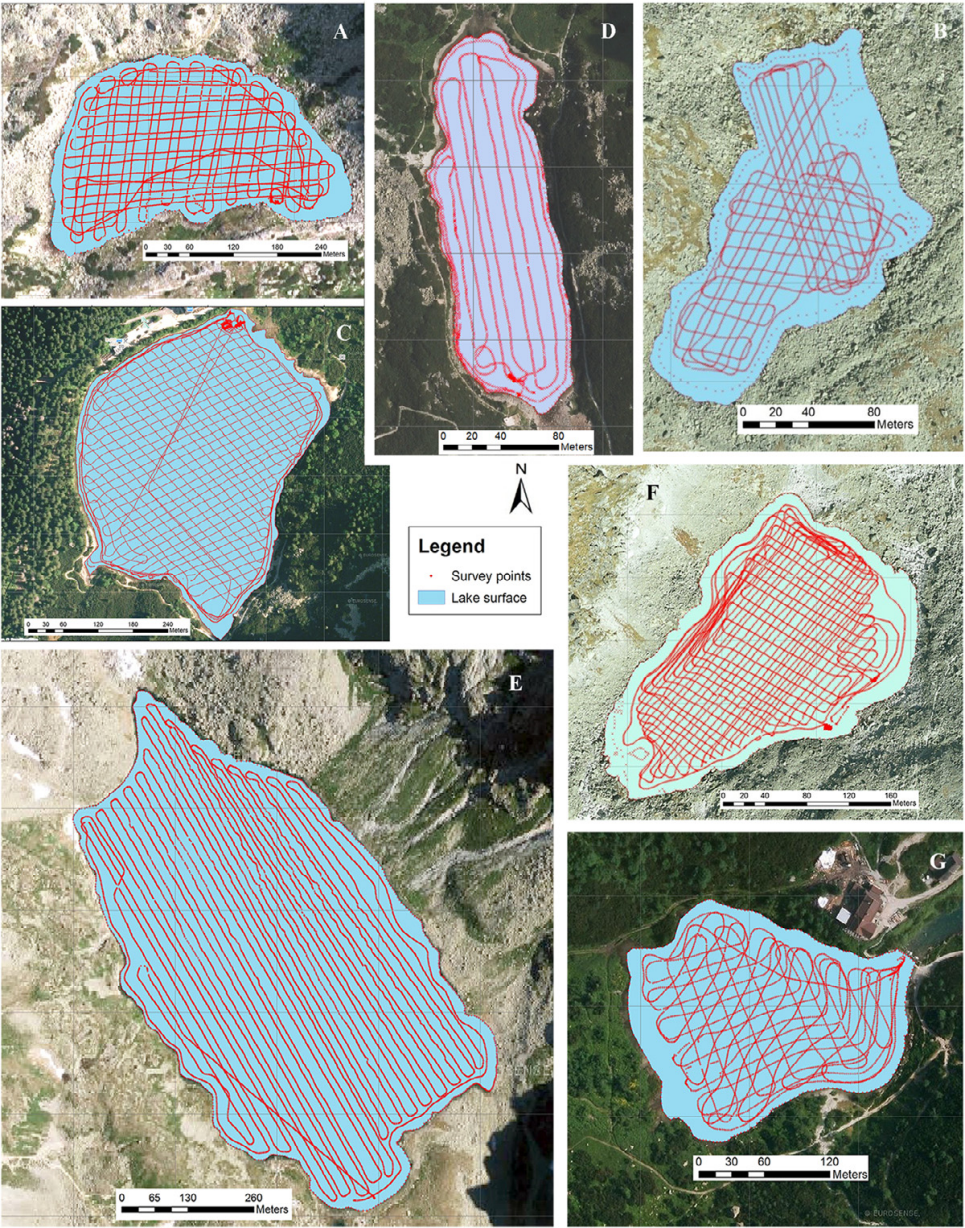
Survey track lines of the AUV EcoMapper. A – Batizovské pleso; B – Nižné Wahlenbergovo pleso; C – Popradské pleso; D - Velické pleso; E – Veľké Hincovo pleso; F – Vyšné Wahlenbergovo pleso; G - Zelené pleso Kežmarské.

**Fig. 5:**
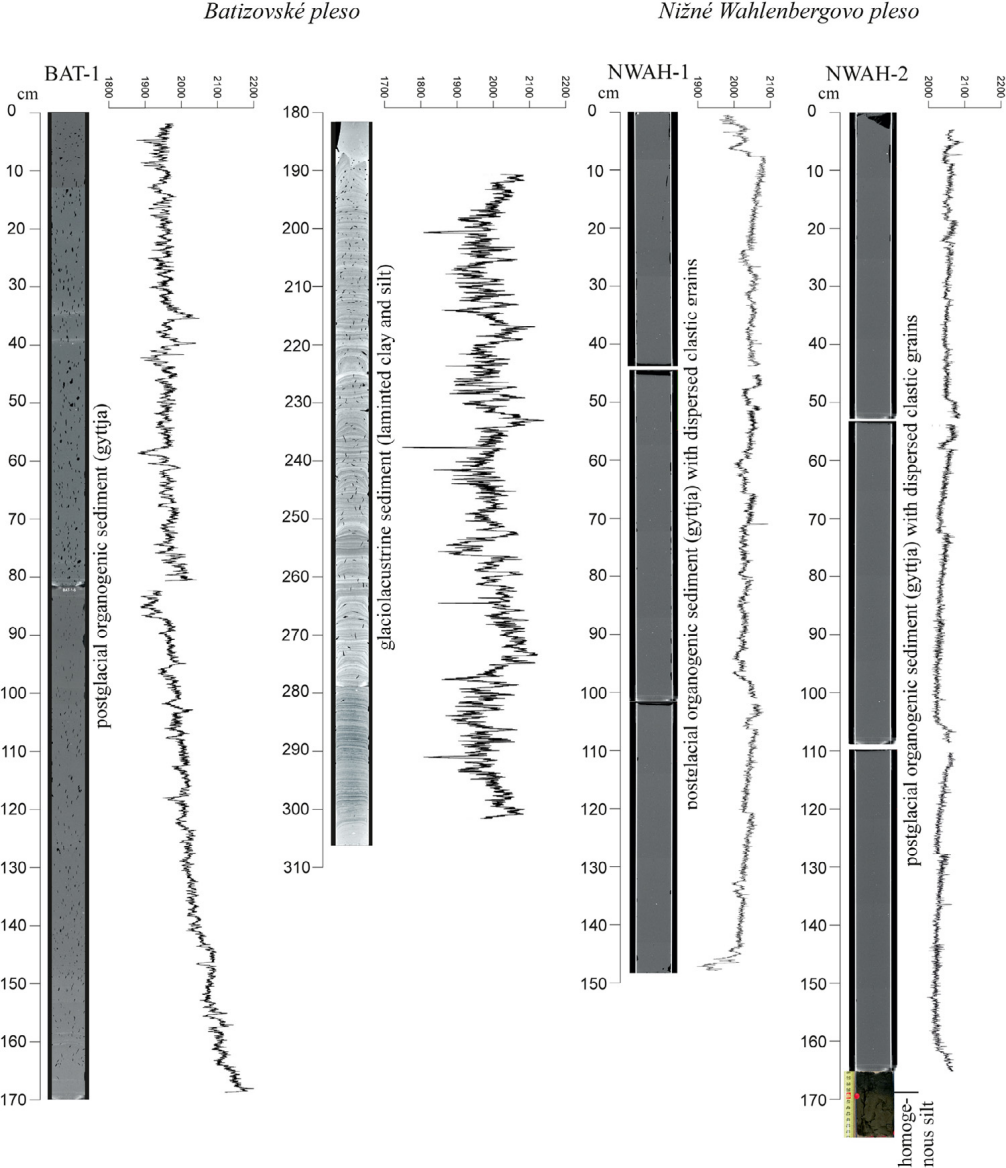
CT scans, lithological, chronostratigraphic interpretation and CT-number values of the cored sedimentary infill of Batizovské pleso and Nižné Wahlenbergovo pleso. Homogenous grey silt (0.07 m) were identified only in the lowermost part of the core NWAH-2 (optic image). It comes from the drill crown therefore it is used for lithological purpose only.

**Fig. 6:**
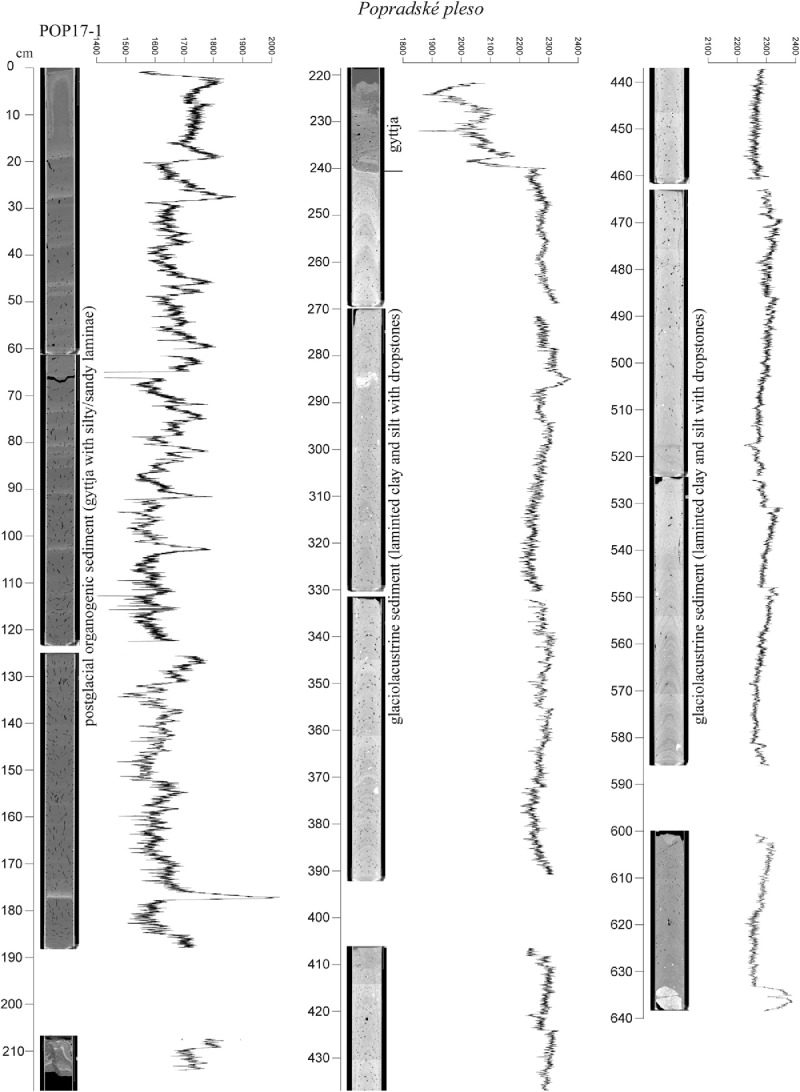
CT scans, lithological, chronostratigraphic interpretation and CT-number values of the cored sedimentary infill of Popradské pleso, core POP17-1.

**Fig. 7:**
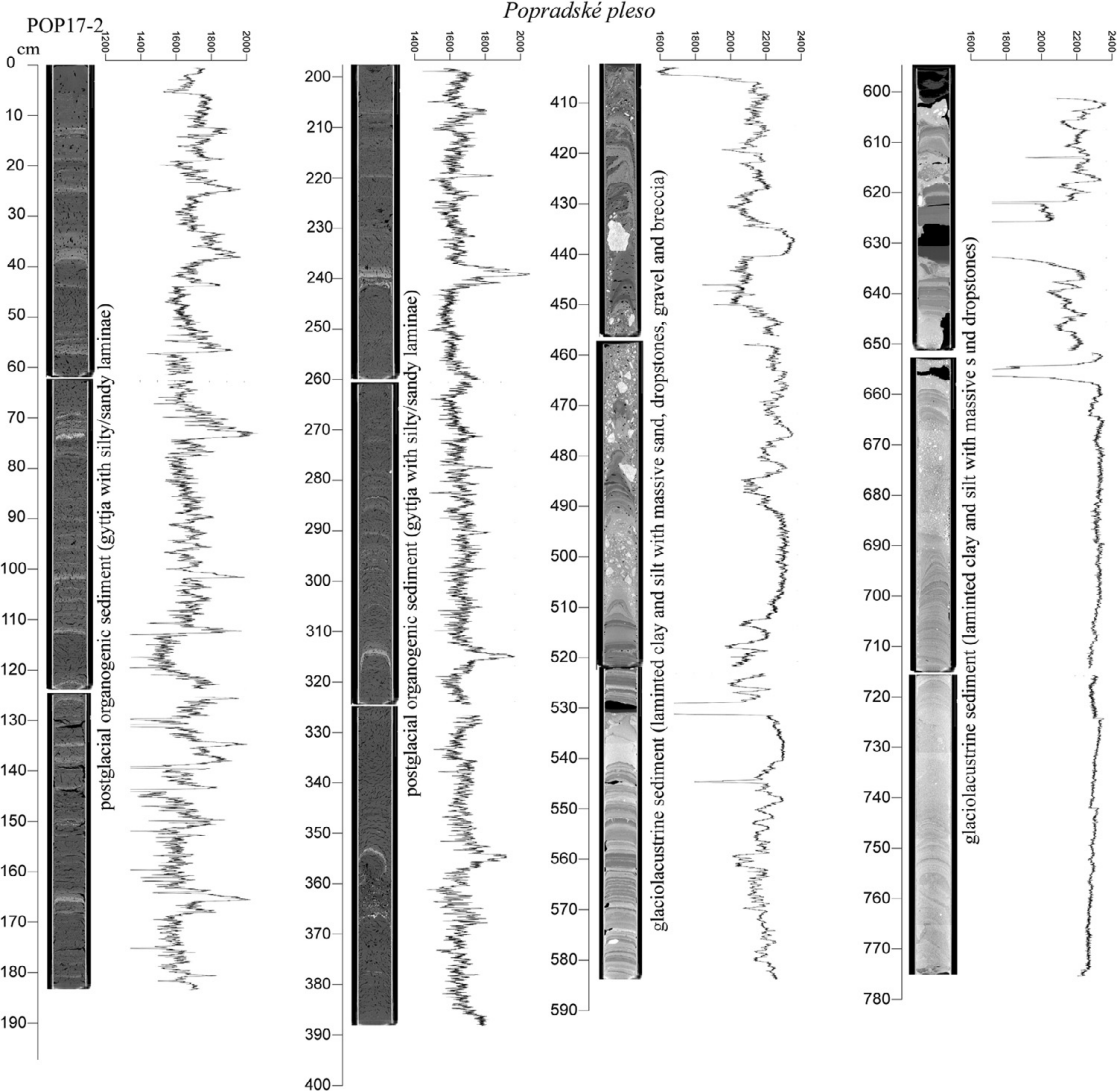
CT scans, lithological, chronostratigraphic interpretation and CT-number values of the cored sedimentary infill of Popradské pleso, core POP17-2. A higher amount of the clastic laminae in the section 0–180 cm than in section 200–388 cm can reflect prograding delta of the Ľadový potok brook or intensification of weathering and subsequent higher clastic input in younger period.

**Fig. 8:**
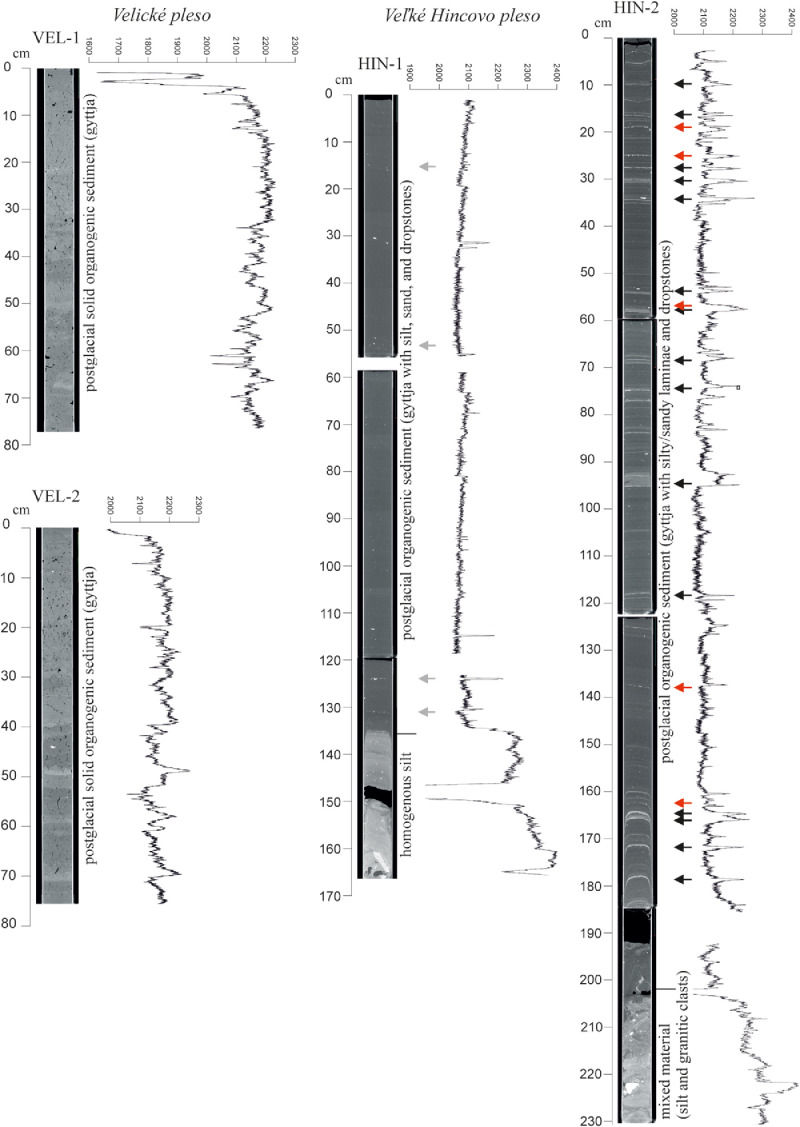
CT scans, lithological, chronostratigraphic interpretation and CT-number values of the cored sedimentary infill of Velické pleso and Veľké Hincovo pleso. The fine and coarse clastics in the deepest part (core HIN-2) concentrate in laminae as a combine effect of rockfalls (red arrow) and debris flow activity (black arrow) while the gyttja of the core HIN-1 contains only dispersed coarse clastics (gray arrow).

**Fig. 9:**
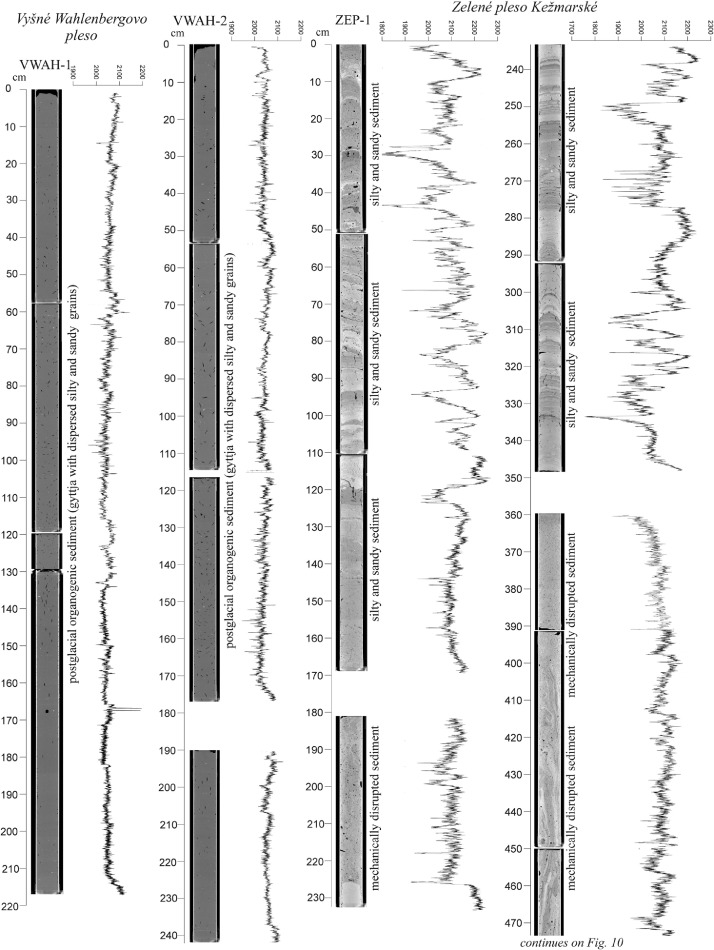
CT scans, lithological, chronostratigraphic interpretation and CT-number values of the cored sedimentary infill of Vyšné Wahlenbergovo pleso and Zelené pleso Kežmarské. The exclusively clastic sedimentation in Zelené pleso Kežmarské in comparison to other Tatra Mts. lakes results from fluvial activity, intensive mass movement processes, and active bottom springs causing permanent mixing of the deposit.

**Fig. 10:**
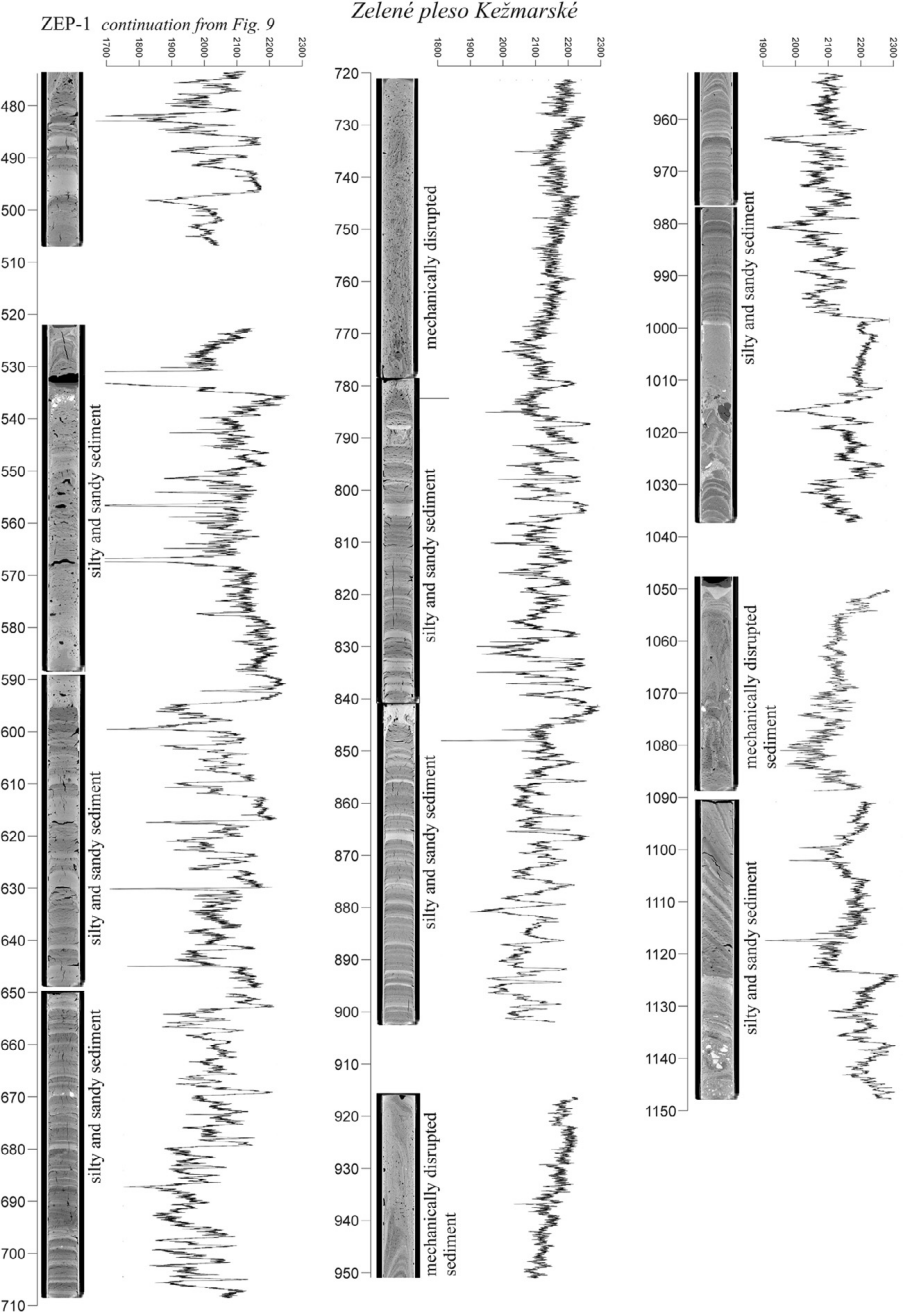
CT scans, lithological, chronostratigraphic interpretation and CT-number values of the cored sedimentary infill of Zelené pleso Kežmarské. CT scanning shows the disturbed and completely mixed sediment in certain segments of the core ZEP-1 which were not visible by optic observations.

**Fig. 11:**
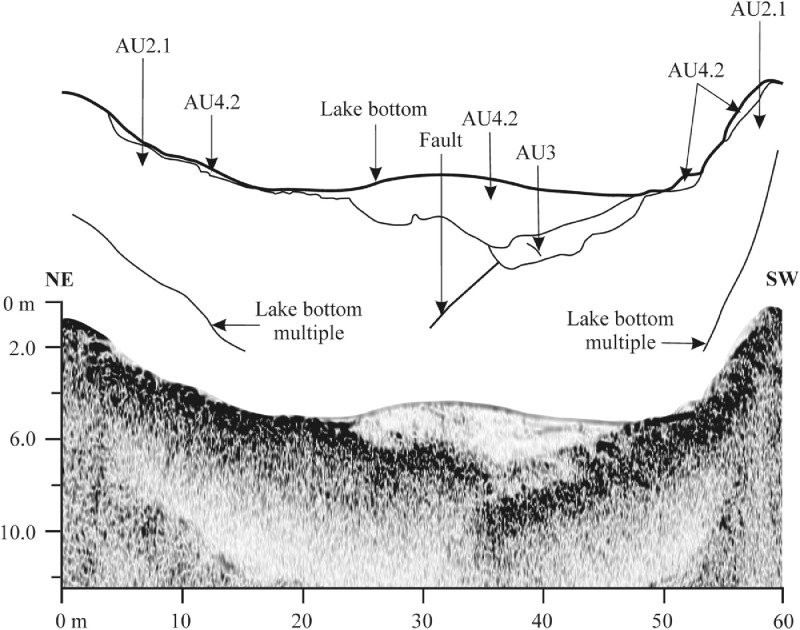
Sub-bottom profile H-H′ of Batizovské pleso showing the body of gyttja with sand admixture and fault on SE margin. For location of the profile see Fig. 3.

**Fig. 12:**
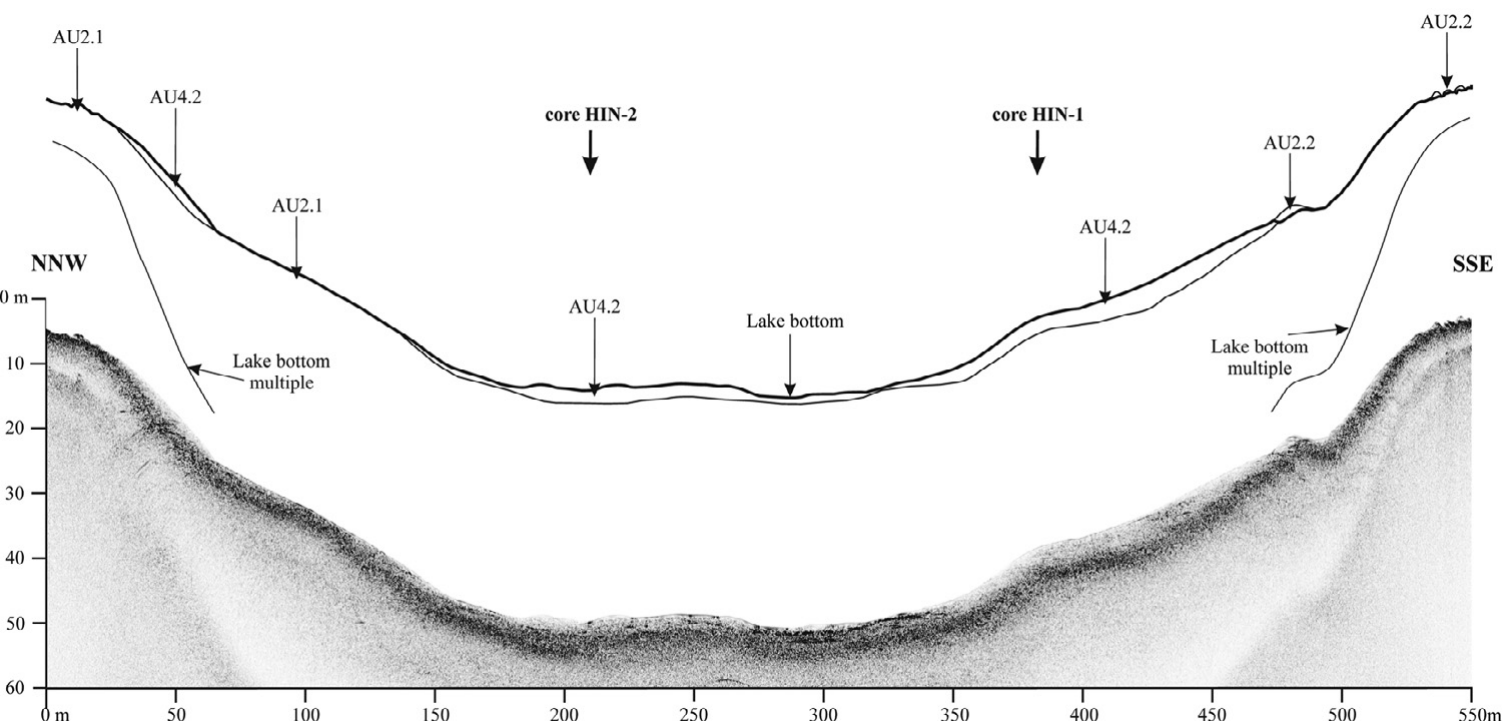
Sub-bottom profile A-A′ of Veľké Hincovo pleso showing the gyttja infill on glacigene deposits. For location of the profile see Fig. 3.

**Table 1:** Missions and acquired sub-bottom sonar (SBS) and AUV EcoMapper data (AUVEM).

		SBS				AUVEM			
Lake	Geographic coordinations	Longitudal profiles	Transversal profiles	Interpreted profile	Total length of sonar profiles [m]	No. of measured points	No. of waypoints	Total length of survey [m]	Duration [min]
**Batizovské pleso**	N49°09′07.46″ E20°07′47.49″	A-E	F-O	C-C′, H-H′	2,193	7,886	122	7,493	121

**Nižné Wahlenbergovo pleso**	N49°09′32.78″ E20°01′31.84″	A-H	1-10	F-F′	1,613	3,973	68	4,100	66

**Popradské pleso**	N49°09′12.47″ E20°04′47.17″	1-11	A-K	H-H′	4,313	18,656	259	14,626	244

**Velické pleso**	N49°09′27.58″ E20°09′21.18″	A-C	D-L	I-I′	1,278	2,503	65	4,102	59

**Veľké Hincovo pleso**	N49°10′44.03″ E20°03′34.73″	A-F	1-8	A-A′, C-C′, 3-3′	5,599	17,417	148	18,771	282

**Vyšné Wahlenbergovo pleso**	N49°09′51.19″ E20°01′33.55″	A-I	1-11	7-7′	3,338	13,065	132	11,497	186

**Zelené pleso Kežmarské**	N49°12′34.44″ E20°13′13.79″	A-D	E-J	D-D′	1,046	4,843	208	4,843	103

Presented bathymetry datasets have been gathered by AUVEM equipped with a single beam echo sounder (SBES) (Table 1). Despite new surveying methods and technologies the SBES is still commonly used in scientific research especially for remote locations and shallow water bodies. In addition, a combination of GNSS Stonex S9II and control unit Ashtech Mobile Mapper 100 was used for the data collection of the shore lines and the water surface altitude.

As the SBES measures the water depth only vertically under the SBES, an interpolation is necessary across areas with no data coverage. The interpolation methods take into account surrounding datasets and estimate missing data using different mathematical interpolations [10]. The outcomes of the bathymetric survey deliver the rare results important for evaluating the sedimentary process and for understanding the ecology and hydrology of mountain glacial lakes. The datasets can serve as a reference for future investigations of the lake dynamics.

**Fig. 13:**
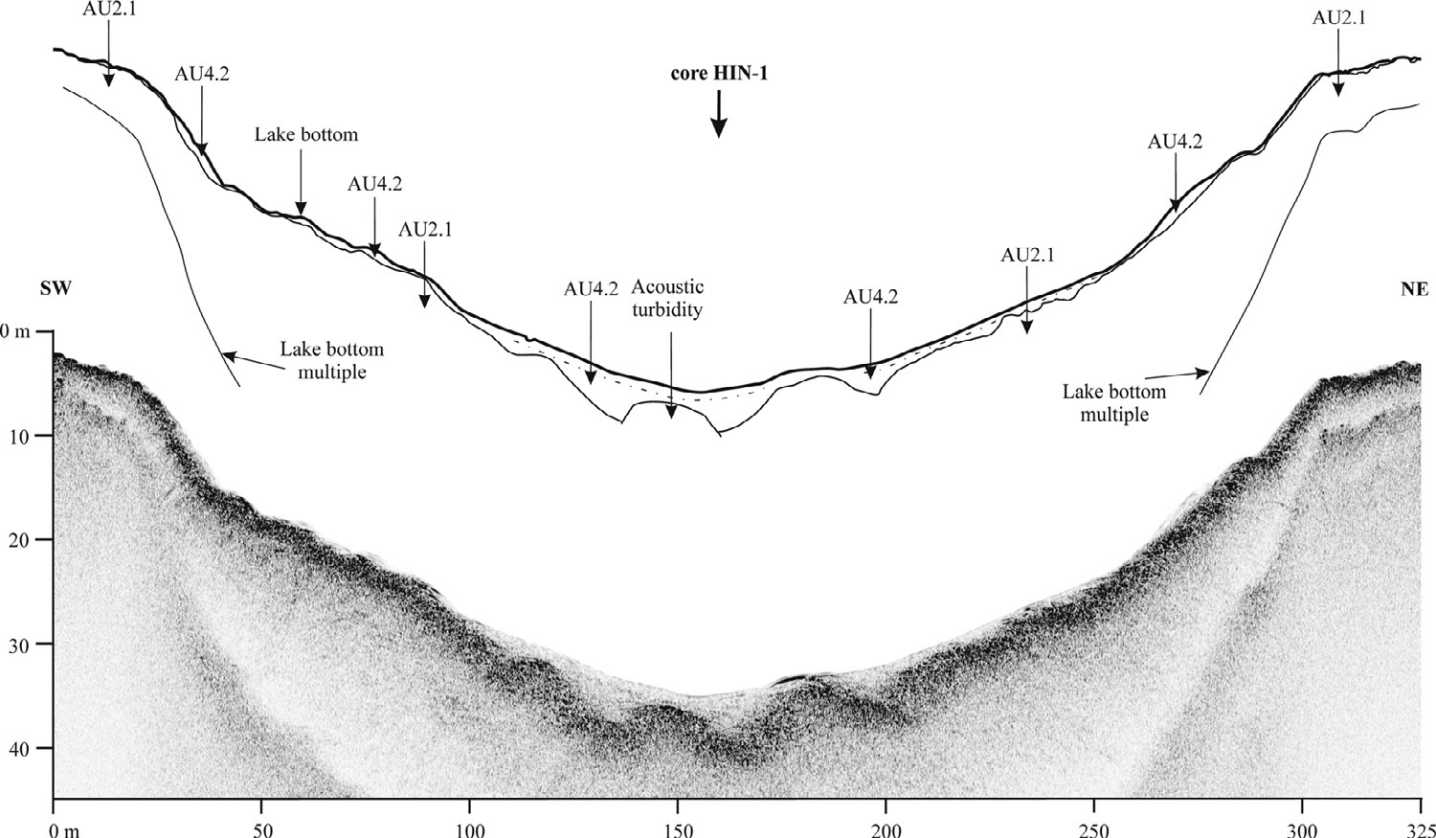
Sub-bottom profile 3-3′ of Veľké Hincovo pleso showing the gyttja infill on glacigene deposits. For location of the profile see Fig. 3.

## CRediT authorship contribution statement

**Ramachandran Dhavamani:** Conceptualization, Writing – original draft, Methodology, Formal analysis, Investigation. **Radovan Pipík:** Conceptualization, Writing – original draft, Methodology, Investigation, Supervision. **Valentín Sočuvka:** Writing – original draft, Methodology, Formal analysis, Investigation. **Juraj Šurka:** Investigation, Methodology. **Dušan Starek:** Investigation, Conceptualization. **Rastislav Milovský:** Investigation, Conceptualization. **Peter Uhlík:** Investigation. **Marina Vidhya:** Resources. **Lucia Žatková:** Resources. **Pavol Král:** Resources, Investigation.

## Declaration of Competing Interest

The authors declare that they have no known competing financial interests or personal relationships which have or could be perceived to have influenced the work reported in this article.

## Appendix Supplementary materials

SM1 Underwater video showing the large conic structure and its morphology, Zelené pleso Kežmarské. Moving deposit documents the activity of these springs and mixing of the sedimentary particles. Image, video 1

Image, application 2
