# Supplementary figures and images for: Glacial and postglacial sedimentary infill in Slovakian High Tatra Mts. lakes: Acoustic survey and lithological data
Source: Data Brief. 2021 Nov 27;40:107644. doi: 10.1016/j.dib.2021.107644 (PMC8713125; doi:10.1016/j.dib.2021.107644)

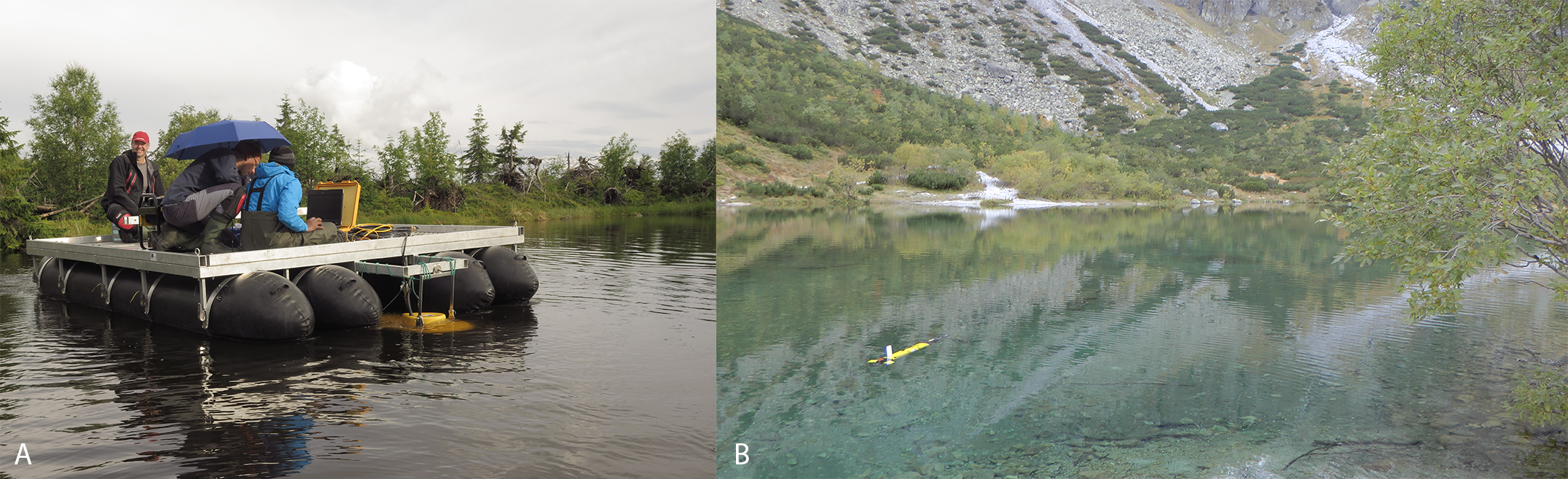

Supplement: Supplementary file 2 [file mmc2.zip › mmc2/DiB Fig 2 SBS and AUVEM in work.tif]

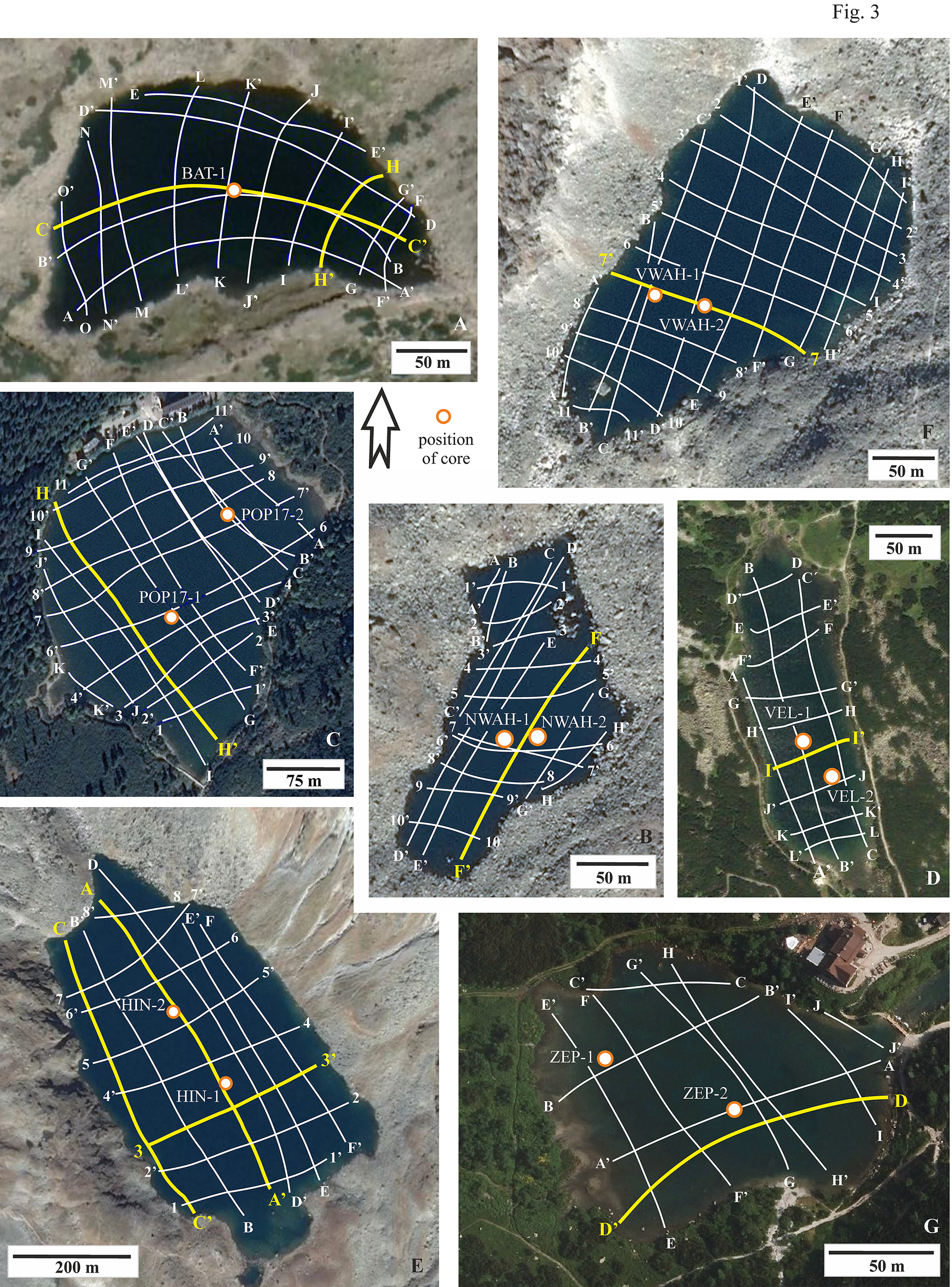

Supplement: Supplementary file 2 [file mmc2.zip › mmc2/DiB Fig 3 SBS track lines.tif]

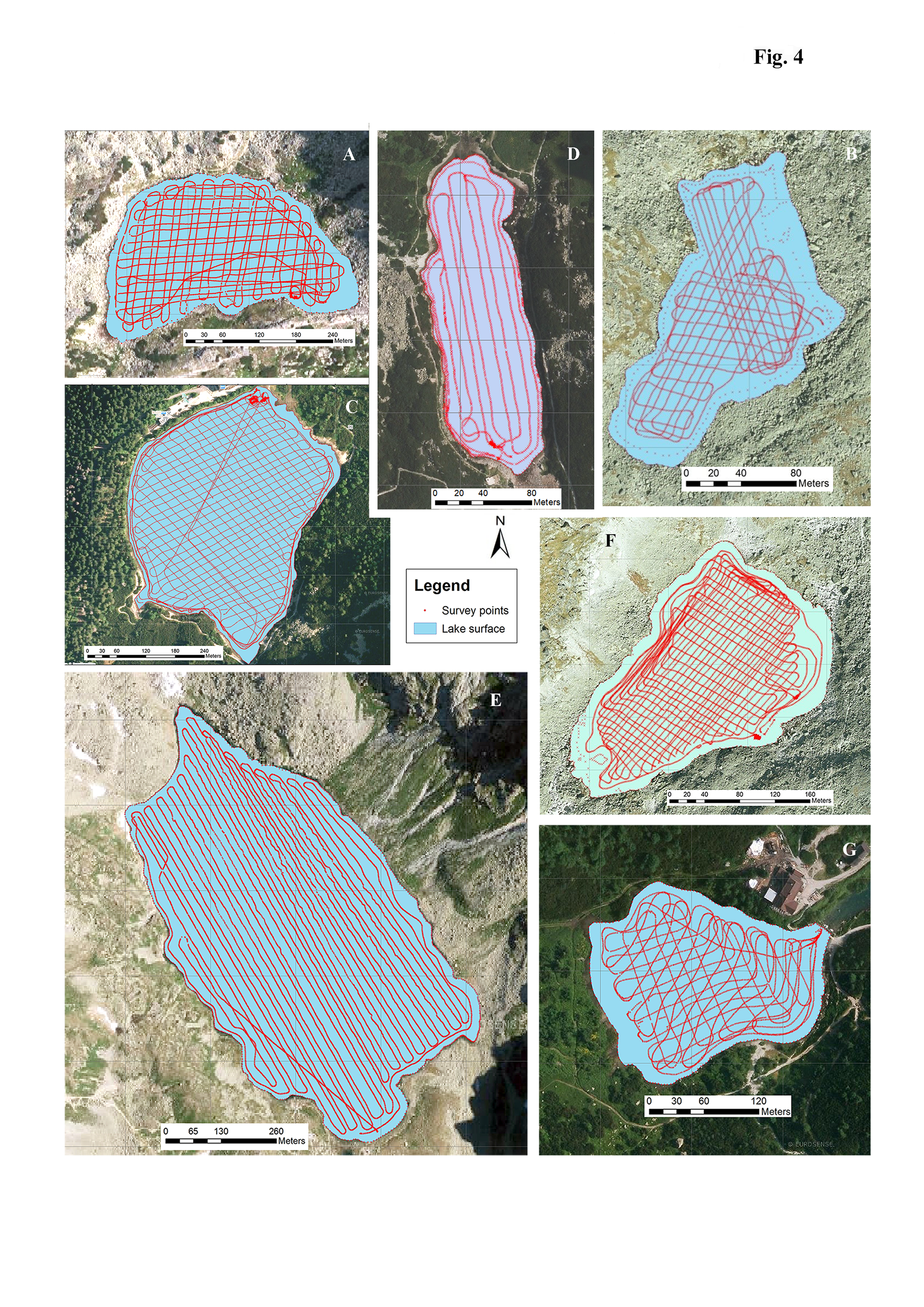

Supplement: Supplementary file 2 [file mmc2.zip › mmc2/DiB Fig 4 AUVEM mission trajectories.tif]
